# Development of Biomarkers Based on DNA Methylation in the *NCAPH2*/*LMF2* Promoter Region for Diagnosis of Alzheimer’s Disease and Amnesic Mild Cognitive Impairment

**DOI:** 10.1371/journal.pone.0146449

**Published:** 2016-01-07

**Authors:** Nobuyuki Kobayashi, Shunichiro Shinagawa, Tomoyuki Nagata, Kazuya Shimada, Nobuto Shibata, Tohru Ohnuma, Koji Kasanuki, Heii Arai, Hisashi Yamada, Kazuhiko Nakayama, Kazuhiro Kondo

**Affiliations:** 1 Department of Virology, The Jikei University School of Medicine, Tokyo, Japan; 2 Department of Psychiatry, The Jikei University School of Medicine, Tokyo, Japan; 3 Division of Molecular Genetics, The Jikei University School of Medicine, Tokyo, Japan; 4 Department of Psychiatry, Juntendo University School of Medicine, Tokyo, Japan; University of Leipzig, GERMANY

## Abstract

From the standpoint of early interventions for dementia, a convenient method of diagnosis using biomarkers is required for Alzheimer’s disease (AD) in the early stage as well as amnesic mild cognitive impairment (aMCI). Focusing on differences in DNA methylation due to AD and aMCI, in the present study, we first conducted genome-wide screening, measuring blood DNA methylation levels by the Illumina Infinium HD Methylation Assay in 3 small age-and gender-matched groups consisting of 4 subjects each: normal controls (NC), aMCI and AD. The genome-wide analysis produced 11 DNA methylation loci that distinguished the 3 groups. For confirmation, we increased group sizes and examined samples by pyrosequencing which revealed that DNA methylation in the *NCAPH2*/*LMF2* promoter region was significantly decreased in the AD (n = 30) and aMCI (n = 28) groups as compared to the NC group (n = 30) (*P* < 0.0001, ANCOVA). No association was found between methylation levels and *APOE* genotype. *NCAPH2*/*LMF2* methylation levels were considered to potentially be a convenient and useful biomarker for diagnosis of AD and aMCI.

## Introduction

Alzheimer’s disease (AD) is a neurodegenerative disease characterized by memory deficit, visuospatial dysfunction, attention deficit and executive dysfunction [1, 2]. The disease slowly progresses through a prodromal phase to the stage of amnesic MCI (aMCI) when symptoms are manifested. Diagnosis at the stage of early AD or aMCI and early interventions will delay its later progression, raising QOL for patients and their caregivers and improving the prognosis [3]. However, at present, not nearly enough patients are examined at specialist medical institutions at the early stage [4]. Therefore, the discovery of a convenient method of diagnosis using biomarkers would increase early stage interventions and aid disease prevention.

According to the amyloid cascade theory or the tau theory, early diagnosis of AD through measurement of amyloidβ (Aβ) or tau in the brain using positron-emission tomography (PET) or cerebrospinal fluid (CSF) assays has been studied [3, 5, 6]. While the effectiveness of amyloid PET in the early diagnosis of aMCI and AD has been reported [7, 8], very few institutions conduct PET imaging because of the cost and time involved. Also, due to the invasiveness of spinal puncture, it is not recommended for the diagnosis of aMCI or AD [9]. There is therefore a need to develop convenient new blood biomarkers.

In recent years, it has been reported that DNA methylation levels in the brain increase with age [10, 11] and that in AD there are differences among several genes in brain DNA regarding methylation [12–15]. However, no association between AD and global DNA methylation was observed [16]. Therefore, we thought that methylation of DNA at specific locations in certain genes could potentially be a convenient and useful diagnosis biomarker.

Regarding blood DNA, we previously reported that there was significantly greater methylation in the brain derived neutrophic factor (BDNF) promoter region of AD patients as compared with controls [17]. However, we were unable to clearly distinguish the 2 groups based on this location. Therefore, our objective in the present study is to find a new blood biomarker that could be used to diagnose AD and to distinguish it from aMCI.

With age- and sex-matched normal controls (NCs), aMCI patients and AD patients as our subjects, we first investigated blood DNA genome-wide for candidate loci with variation in methylation levels that could be used to clearly distinguish between the 3 groups. We also examined associations between DNA methylation and mini-mental state examination (MMSE) and frontal assessment battery (FAB) scores which reflect cognitive impairment. In addition, we examined an association betweeen DNA methylation and *APOE* genotype, the greatest genetic risk factor of AD.

## Materials and Methods

### Ethics statement

The study was approved by the Ethics Committee of the Jikei University School of Medicine and Juntendo University School of Medicine, and written informed consent was obtained from all subjects. For participants whose capacity to consent was compromised, caregivers who were the spouse or a relative within the second degree consented on their behalf. For patients with aMCI or AD who were able to sign the consent form, if there was any possibility of them forgetting that they had consented to participation, written informed consent was obtained from both patients and caregivers.

### Subjects

From among consecutive memory clinic outpatients visiting the Jikei University Hospital (Tokyo) or the Jikei University Kashiwa Hospital (Kashiwa City, Chiba Prefecture), 30 patients with AD and 28 patients with aMCI were enrolled. The 30 NCs were recruited from Juntendo University Hospital (Tokyo) [18, 19]. AD was diagnosed based on the US National Institute of Neurological and Communicative Disorders and Stroke and the Alzheimer's Disease and Related Disorders Association (NINCDS-ADRDA) criteria and aMCI by the criteria defined by Peterson [20, 21]. aMCI included both amnestic MCI-single domain- and MCI-multiple domain-type subjects. The neuropsychiatric symptoms for AD were assessed based on information from a structured interview with each patient’s caregiver by geriatric psychiatrists using the behavioral pathology in Alzheimer’s disease (Behave-AD) scale [22]. Two neuropsychological tests, MMSE and FAB, were administered to AD and aMCI patients by a clinical psychologist [23, 24]. MMSE is widely used for assessment of cognitive function and FAB is an effective tool for assessment of executive function. Thus MMSE and FAB differ in their focuses. We previously reported that FAB was effective for distinguishing between AD and MCI [25] and in the present study, we felt that it would be useful to employ 2 scales with different focuses for this purpose. NCs did not satisfy the clinical criteria for AD or MCI; however, if they had past history of treatment for other psychiatric disorders, they were excluded. Genomic DNA was extracted from white blood cells using a standard method [18, 19].

### *APOE* genotyping

*APOE* genotypes (rs429358 and rs7412) were determined by allelic discrimination on an Applied Biosystems 7300 real-time PCR System (Applied Biosystems). The amplifications were performed in duplicate in a total volume of 25 μl containing 12.5 μl of 2✕ TaqMan Universal PCR Master Mix II, No AmpErase UNG, 0.625 μl of 40✕ Primer and TaqMan Probe dye mix (assay ID C____3084793_20 and C ____904973_10) (Applied Biosystems), 1.0 μl of the genomic DNA, and 10.875 μl of PCR-grade water. The thermal profile was 50°C for 2 min and 95°C for 10 min, followed by 40 cycles of 92°C for 15 sec and 60°C for 1 min. Data analysis used Sequence Detection Software version 1.4 (Applied Biosystems).

### DNA methylation profiling

Bead array analysis was conducted according to the Manual Protocol for the Illumina Infinium HD Methylation Assay. Briefly, using EZ DNA Methylation Kit (Zymo Research Inc.), bisulfite conversion was performed on each 1.0 μg genomic sample followed by purification and recovery. After denaturing with alkali, the whole genome was amplified and then fragmented, both processes using enzymes, purified through sedimentation with isopropanol and re-suspended in hybridization buffer. The re-suspended DNA was then heat denatured and applied to HumanMethylation450 BeadChip, and hybridization conducted for around 23 h in a hybridization oven. Next, the Bead Chip was washed with buffer, and then, a labeled nucleotide was incorporated at the end of the probe. The Bead Chip was stained using a fluorescent marker antibody to the incorporated nucleotide. Next, it was washed in buffer and after coating and drying, fluorescence images were obtained using iScan Control Software ver. 3.2.45 (Illumina, Inc.). Background Subtraction and normalization to Internal Controls was then performed using GenomeStudio ver. 2011.1/Methylation Module ver. 1.9.0 (Illumina, Inc.).

### Pyrosequencing

**Fig 1:**
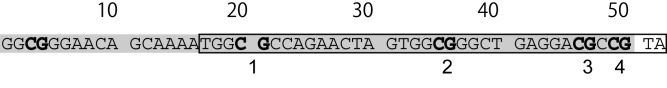
Part of Sequence *NCAPH2*/*LMF2* Promoter Region from Pyrosequencing. The gray area indicates the source sequence of the Target id: cg25152348. The open box indicates the target sequence of pyrosequencing.

Pyrosequencing was conducted for 4 of 5CpGs including the Target id: cg25152348 according to the standard protocol (Fig 1). Briefly, 500 ng of each genomic DNA sample was bisulfite-converted using EpiTect Plus DNA Bisulfite Kit (Qiagen, Inc.), purified and recovered. Amplifications were performed in a total volume of 25 μl containing 12.5 μl of 2✕ PyroMark PCR Master Mix, 2.5 μl of 10✕ CoralLoad Concentrate, 0.5 μl of 10 μM forward primer (5′-GTTTAAATTGGTGGTAGTTTAAAGT-3′), 0.5 μl of 10 μM biotin labeled reverse primer (5′- -biotin-TCCACCTCCCAATTCTTAATAAAATC-3′), 1.0 μl of the bisulfited DNA, and PCR-grade water. The thermal profile was 95°C for 15 min, 45 cycles of 94°C for 30 sec, 56°C for 30 sec and 72°C for 30 sec, followed by a final extension at 72°C for 10 min. Single-stranded DNA templates were prepared from 20 μL of the biotinylated PCR product using Streptavidin-coated Sepharose beads (Streptavidin Sepharose High Performance. GE Healthcare, Inc.) and 0.3 μM sequence primer (5′-TTTGGGAGGGAATAGTAAAA-3′) annealed to this. Primed templates were sequenced using PyroMark Q24 System (Qiagen, Inc.) and the assay setup generated using PyroMark Q24 Application Software 2.0 (Qiagen, Inc.).

### Statistical Analysis

ANOVA was used to compare background characteristics of aMCI, AD, and NC subjects, other than gender and *APOE* genotype which were compared using the chi-squared test. The unpaired t test was used to compare duration of disease, age at onset, MMSE and FAB scores between the aMCI and AD groups. Spearman's rank correlation coefficients were used to investigate correlations between individual background characteristics and DNA methylation levels. Also, correlations between Illumina Infinium HD Methylation Assay and pyrosequencing methylation levels were examined using Pearson correlation coefficients. The unpaired t test was used to compare methylation levels between 2 groups. Differences in methylation levels among the 3 groups were assessed by analysis of covariance (ANCOVA) with age as a covariate. *P* < 0.05 was considered statistically significant.

Statistical analysis was conducted using Microsoft Excel for Mac 2011 (Microsoft Corporation, Inc.), SPSS Statistics 21 for windows (IBM, Inc) and Prism 6 for Mac OS X (GraphPad Software Inc.).

## Results

### Patient Characteristics

There were no significant differences among the NC, aMCI, and AD groups regarding age and gender, and none were observed for duration of disease and age at onset between the aMCI and AD groups. However, MMSE and FAB scores were significantly lower in the AD group than in the aMCI group. There was a high prevalence of *APOE* allele 4 in the AD group (Table 1).

**Table 1:** Subject Characteristics (mean ± S.E.M.).

	NC (n = 30)	MCI (n = 28)	AD (n = 30)	*P*
Age	70.5 ± 1.0	72.0 ± 0.9	71.8 ± 0.9	0.452
Female: male (%)	60.0: 40.0	53.6: 46.4	53.3: 46.7	0.842
Duration of disease (months)	-	26.5 ± 4.4	28.2 ± 3.5	0.772
Age at onset	-	69.8 ± 0.9	69.4 ± 1.0	0.790
MMSE score	-	27.2 ± 0.4	18.5 ± 1.0	0.001 **
FAB score	-	14.8 ± 0.4	12.0 ± 0.7 #	0.000 ****
*APOE* allele ε2	3	1	0	0.551
*APOE* allele ε3	46	43	35	0.144
*APOE* allele ε4	11	12	25	0.036 *

Age was analyzed by one-way ANOVA. Sex ratios were analyzed by the Chi-Square test. Other values were analyzed by the unpaired t test.

* *P* < 0.05

** *P* < 0.01

**** *P* < 0.0001

# n = 23

### Associations of DNA Methylation in Target Loci

**Fig 2:**
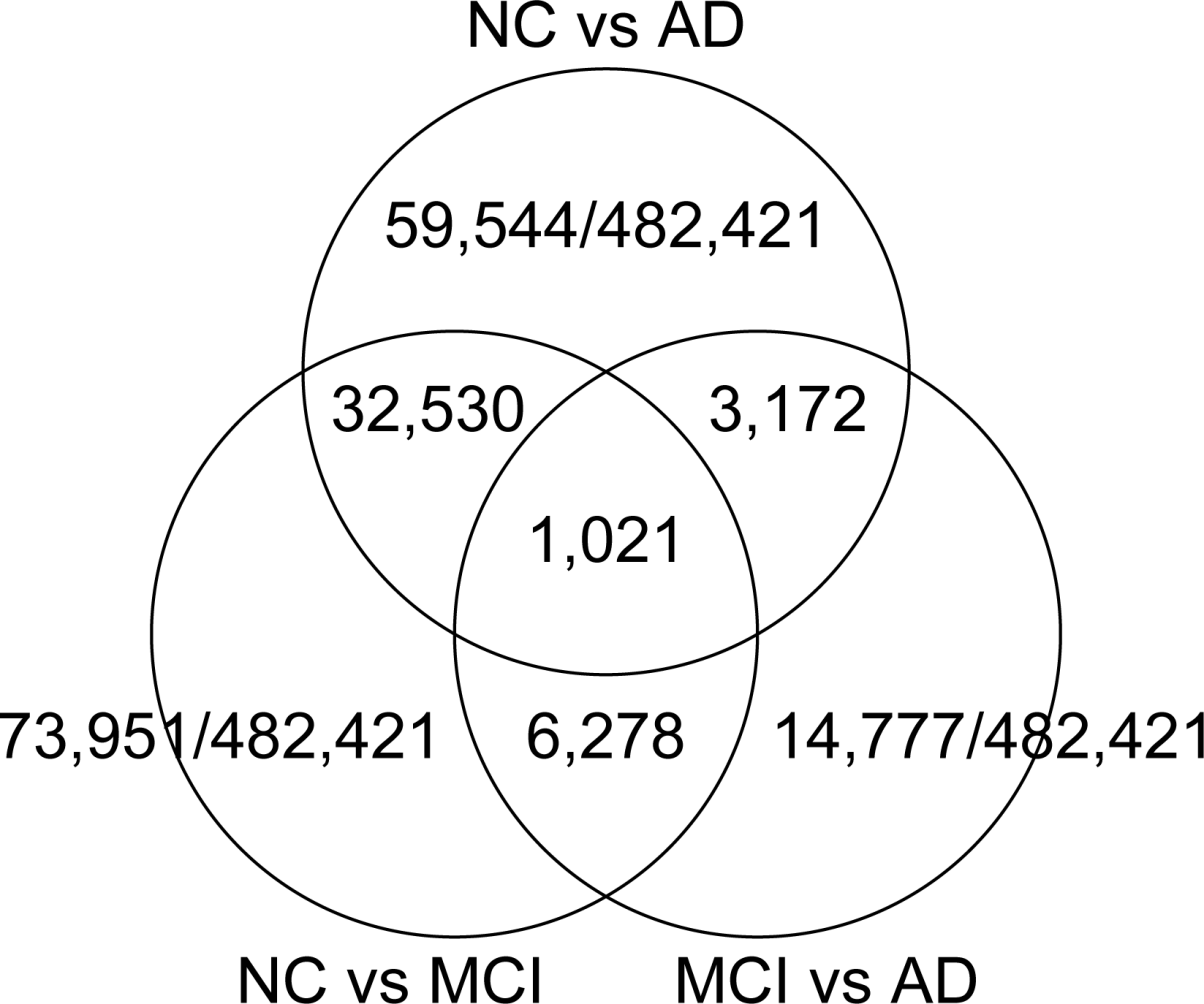
Two-group Differences in Methylation Levels amongAD, aMCI and NC Groups. Methylation levels obtained using HumanMethylation450 BeadChip were compared between NC, aMCI or AD group (n = 4) and one other group. The numbers of loci having significant differences are indicated.

For the 4 subjects in each of the 3 groups (AD, aMCI and NC analyzed by HumanMethylation450 BeadChip) we analyzed 485777 loci, of which 482421 were CpG loci. We next determined candidate loci for which there were significant differences between the AD, aMCI and NC groups individually and one other group (Fig 2). Of 1021 loci for which there were significant, common 2-group differences for all 3 groups, 156 were connected with progression of methylation and symptoms, and 129 of them were loci in genetic regions that had been assigned a USC ACCESSION Number. Further, in areas neighboring transcription start sites (TSS), 1st Exon, and 5'UTR where methylation levels would have a direct influence on gene expression and be low, there were 57 loci (S1 Table) [26, 27]. Among them, there were 11 loci with CpG islands (Table 2).

**Table 2:** 11 Candidate Loci produced by the Illumina Infinium HD Methylation Assay.

Target ID	UCSC REFGENE NAME	UCSC REFGENE ACCESSION	UCSC REFGENE GROUP
cg01756799	*COASY;COASY;COASY;COASY;COASY*	NM_025233;NM_001042529;NM_001042532;NM_001042530;NM_001042531	1stExon;Body;Body;Body;5'UTR
cg06695761	*SGCE;PEG10;SGCE;PEG10;SGCE*	NM_001099401;NM_015068;NM_001099400;NM_001040152;NM_003919	TSS1500;5'UTR;TSS1500;5'UTR;TSS1500
cg08727202	*MPST;MPST;TST;MPST;MPST*	NM_001013436;NR_024038;NM_003312;NM_001130517;NM_021126	TSS1500;TSS1500;Body;TSS1500;TSS1500
cg09898695	*SPINT1;SPINT1;SPINT1*	NM_003710;NM_001032367;NM_181642	TSS1500;TSS1500;TSS1500
cg13523072	*COASY;COASY;COASY;COASY;COASY*	NM_025233;NM_001042529;NM_001042532;NM_001042530;NM_001042531	1stExon;Body;Body;Body;5'UTR
cg13947830	*MIB2;MIB2;MIB2;MIB2;MIB2;MIB2*	NM_001170687;NM_001170688;NM_080875;NM_001170689;NM_001170686;NR_033183	Body;Body;Body;5'UTR;Body;Body
cg19205533	*RERG*	NM_032918	5'UTR
cg23779106	*DUSP12*	NM_007240	1stExon
cg25152348	*NCAPH2;LMF2;NCAPH2;NCAPH2;NCAPH2*	NM_152299;NM_033200;NM_152299;NM_014551;NM_014551	1stExon;TSS1500;5'UTR;5'UTR;1stExon
cg26812418	*CPE*	NM_001873	TSS200
cg27173717	*MFSD2A;MFSD2A*	NM_032793;NM_001136493	TSS1500;TSS1500

Table indicates the target ID assigned by the Illumina Infinium HD Methylation Assay as well the gene name, accession number and gene group registered in UCSC.

### Associations between Methylation Levels and MMSE, FAB Scores

Negative correlations were observed between methylation levels of CpG islands in the 11 loci and MMSE score for the *COASY*, *SPINT1*, *RERG* and *NCAPH2*/*LMF2* gene regions, and there were negative correlations with FAB score for the *COASY* and *NCAPH2*/*LMF2* gene regions (Table 3). Methylation levels were significantly correlated with both MMSE and FAB scores in the *COASY* (Target ID: cg01765799) and *NCAPH2*/*LMF2* (Target ID: cg25152348) gene regions. The correlation for the *NCAPH2*/*LMF2* gene region was stronger and the *P* value was lower (Table 3). A positive correlation was observed between MMSE and FAB scores (*ρ* = 0.46, *P* = 0.001; Spearman’s rank correlation coefficient).

**Table 3:** Spearman’s Rank Correlations between Methylation Levels and MMSE, or FAB Scores.

Target ID	GENE NAME	MMSE			FAB		
		*ρ*	*P*	n	*ρ*	*P*	n
cg01756799	*COASY*	-0.76	0.031 *	8	-0.88	0.021 *	6
cg06695761	*SGCE/PEG10*	-0.66	0.076	8	-0.70	0.123	6
cg08727202	*MPST/TST*	-0.65	0.083	8	-0.76	0.080	6
cg09898695	*SPINT1*	-0.73	0.040 *	8	-0.40	0.439	6
cg13523072	*COASY*	-0.49	0.217	8	-0.82	0.046 *	6
cg13947830	*MIB2*	-0.64	0.091	8	-0.40	0.439	6
cg19205533	*RERG*	-0.90	0.002 **	8	-0.52	0.295	6
cg23779106	*DUSP12*	-0.42	0.301	8	0.40	0.439	6
cg25152348	*NCAPH2/LMF2*	-0.89	0.003 **	8	-0.94	0.005 **	6
cg26812418	*CPE*	-0.68	0.062	8	-0.15	0.774	6
cg27173717	*MFSD2A*	-0.64	0.091	8	-0.40	0.439	6

* *P* < 0.05

** *P* < 0.01

### Pyrosequencing of *NCAPH2*/*LMF2* Gene Region

In order to verify CpG methylation levels in the *NCAPH2*/*LMF2* gene region, we conducted pyrosequencing for an AD group, aMCI group and a NC group having 30, 28 and 30 age- and sex-matched subjects, respectively, which included the subjects whose samples were analyzed in the Illumina Infinium HD Methylation Assay (Fig 1). Among the 4 CpGs, analysis revealed significant correlations between methylation levels by pyrosequencing and the Ilumina Infinium HD Methylation Assay for CpG 2–4 (S1 Fig).

No gender difference in methylation levels was observed among CpG 1–4. Although the methylation level in CpG 3 was negatively correlated with age, levels were not correlated with duration of disease or age at onset (S2 Table)

**Fig 3:**
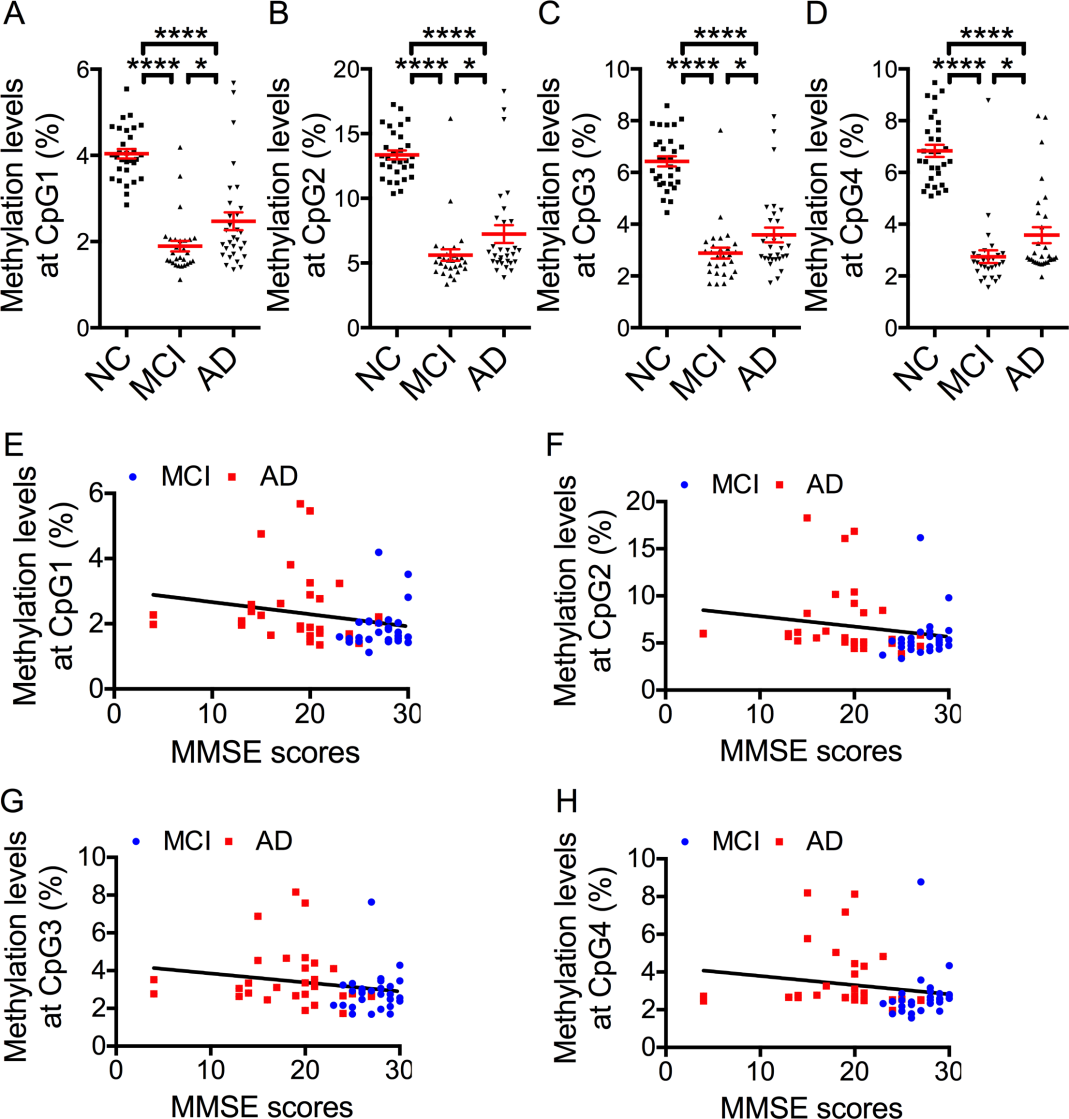
Comparison of Methylation Levels in NC, aMCI and AD Groups. (A-D) Comparison of methylation levels in 3 groups for each CpG. Red horizontal lines are means, error bars indicate S.E.M. * *P* < 0.05, **** *P* < 0.0001. (E-H) Correlations between MMSE score and methylation levels in each CpG. Blue dots indicate aMCI, red dots AD.

Next, with age as a covariate, we compared methylation levels at the 4 CpG sites. Compared with the NC group, methylation levels in the aMCI and AD groups were lower (Fig 3A–3D). Also, methylation levels in the AD group were significantly higher than in the aMCI group (Fig 3A–3D). In addition, there was a significant correlation between methylation levels in the 4 CpGs and MMSE score (*ρ* = -0.32, *P* = 0.013 * (A); *ρ* = -0.29, *P* = 0.026 * (B); *ρ* = -0.26, *P* = 0.048 * (C); *ρ* = -0.26, *P* = 0.049 * (D); * *P* < 0.05, Spearman's rank correlation coefficient) (Fig 3E–3H). However, there was no correlation with FAB score. Furthermore, methylation levels in the *NCAPH2*/*LMF2* gene region were not significantly different between *APOE* allele 4 carriers and non-carriers.

## Discussion

Genome-wide screening of differences in DNA methylation levels related to AD onset in the groups with 4 subjects—AD, aMCI and NC—produced 11 loci that met the conditions: they were in CpG islands, they were in gene areas with UCSC ACCESSION Numbers, differences were in the order NC, aMCI, AD (Table 2). Among loci having correlations with MMSE and FAB scores, we selected the promoter region of the *NCAPH2*/*LMF2* genes for our research because the correlations were the strongest and the *P* value was the lowest (Table 3). However, the results were from small samples and lacked statistical power. To confirm reproducibility, we performed pyrosequencing on samples from an increased number of subjects in each group. As a previous study had found no difference in methylation between sense and antisense directions in pyrosequencing [28], we biotinized the reverse primer.

As the pyrosequencing results were correlated with those of the Illumina Infinium HD Methylation Assay, we considered our initial results to be reasonable (S1 Fig). In the results of pyrosequencing, methylation levels in the *NCAPH2*/*LMF2* gene promoter region were significantly lower in the aMCI and AD groups than in the NC group (Fig 3A–3D).

Thus, after increasing the size of the study populations, methylation rates were not in the order NC, aMCI, AD but NC, AD, aMCI. However, the difference in methylation levels between aMCI and AD was smaller than between NCs and aMCI or AD, and this suggested that as aMCI and AD are similar diseases, methylation levels could be a specific marker in their common neurodegeneration process (Fig 3A–3D). We considered that one reason for this finding was bias due to the small sample size. As the difference for aMCI, was greater, our findings also suggested the possibility that methylation in the *NCAPH2*/*LMF2* gene promoter region might be a powerful biomarker for detecting the disease in the early stage.

Epidemiological research has shown that aMCI does not necessarily develop into AD; cohorts which stayed at the stage of aMCI and those that reverted to normal have been reported [29, 30]. In the present study, although the prevalence of *APOE* allele 4 was higher in the AD group than in the NC group, there was no difference between the aMCI and NC groups (Table 1). So from the genetic background, we could not definitely say that aMCI group subjects would progress to AD. As methylation levels in the *NCAPH2*/*LMF2* gene promoter region were decreased in both the aMCI and AD groups, this marker may reflect some kind of degenerative process in the brain, but not the clinical onset of AD. As a supporting finding, there was no association between methylation levels and *APOE* allele 4, which could mean that they act independently of AD genetic risk factors. However, the results suggest a correlation with age, the greatest risk factor for developing AD (S2 Table). They also suggest that methylation in the *NCAPH2*/*LMF2* gene promoter region is a trigger for aMCI and AD pathology, irrespective of negative genetic factors.

As dementia progressed in our subjects, we speculated that methylation levels would slowly rise towards original levels within a range much lower than that for NC subjects (Fig 3A–3D). One reason that pyrosequencing did not replicate the initial genome wide screening results was thought to be technical limitations of the pyrosequencing procedure. While the Illumina Infinium HD Methylation Assay directly detects methylation levels by means of a probe, with pyrosequencing, amplification by PCR is conducted prior to analysis so there is therefore the possibility of bias due to PCR influencing the results, but the mechanism would be unclear if this were the case. Also the influence of the small sample size cannot be ruled out.

Also, since there was a correlation not with disease duration but with MMSE scores, which reflect cognitive dysfunction, it was doubted whether there would be an association between progress of cognitive dysfunction and methylation levels (Fig 3E–3H, S2 Table). Furthermore, in pyrosequencing, we considered that the lack of a correlation between methylation levels and FAB was because methylation levels reflect an overall decline in cognitive function, which is assessed by MMSE, not a decline in executive function, which is assessed by FAB. The correlation between FAB and methylation levels observed in the initial screening was probably due to the small number of samples and a Type I error arising from the confounding factor of the correlation between MMSE and FAB scores.

However, it is not clear whether differences in methylation levels in the *NCAPH2*/*LMF2* gene region are the cause of cognitive dysfunction in AD, or reflect degeneration in the brain. *NCAPH2* is the gene that codes for non-SMC condensing II complex subunit H2, also known as Condensin-2 complex subunit H2 or chromosome-associated protein H2 (CAP-H2). This protein is a subunit of condensin II and its complexes are associated with mitosis. *LMF2* is the gene that codes for lipase maturation factor 2. It is known to be a paralog of *LMF1* and thought to be connected with the maturation and transport of lipoprotein lipases. As methylation levels in the *NCAPH2*/*LMF2* gene promoter region were decreased in the aMCI and AD groups, we could expect that expression of the *NCAPH2*/*LMF2* genes would be elevated but it is unknown whether this was the case because we did not measure mRNA expression. It is also unknown whether the gene expression of either *NCAPH2* or *LMF2*, or both, is associated with aMCI and AD onset. In future studies, it will be necessary to examine whether enhanced expression due to decreased methylation levels contributes to accumulation of Aβ or tau phosphate and promotes neuron degeneration, or whether methylation levels decrease in accordance with reactivity accompanying neuron degeneration, or whether raising methylation levels of these genes would be a useful therapy in the treatment of aMCI and AD.

As a technical problem of the present study, pyrosequencing was conducted after amplifying bisulfite converted DNA by PCR, so it would be possible for a difference in amplification efficiency between methylated and non-methylated DNA to arise. However, even if there had been a bias due to PCR, our findings still suggest that methylation levels would be a useful diagnosis tool for distinguishing between NCs and aMCI and AD. Also, as this technique uses blood, it can be conveniently performed without having to be in a special location.

Furthermore, while the present research only investigated the *NCAPH2*/*LMF2* gene region, and it lacked statistical power, it did determine several other candidate genes. It might be possible to raise accuracy by measuring methylation levels for these genetic regions as well.

In conclusion, methylation levels in the *NCAPH2/LMF2* gene region were considered to be a convenient and useful biomarker for diagnosing AD and aMCI.

## Supporting Information

S1 Fig Correlation between Methylation Levels Quantified by Illumina Infinium HD Methylation Assay and Pyrosequencing for CpG1 (A), CpG2 (B), CpG3 (C) and CpG4 (D). *r* = -0.54, *P* = 0.069 (A); *r* = -0.61, *P* = 0.037 * (B); *r* = -0.58, *P* = 0.046 * (C); *r* = -0.58, *P* = 0.047 * (D); * *P* < 0.05, Pearson's correlation coefficient.
(TIFF)

S1 Table 57 Candidate Loci Obtained by Illumina Infinium HD Methylation Assay. Table indicates the target ID assigned by the Illumina Infinium HD Methylation Assay as well the gene name, accession number and gene group registered in UCSC.
(DOCX)

S2 Table Correlations between Methylation Level and Age, Duration of Disease or Age at Onset. * *P* < 0.05, Spearman's rank correlation coefficient.
(DOCX)
