# Supplementary figures and images for: Development of Biomarkers Based on DNA Methylation in the NCAPH2/LMF2 Promoter Region for Diagnosis of Alzheimer’s Disease and Amnesic Mild Cognitive Impairment
Source: PLoS One. 2016 Jan 7;11(1):e0146449. doi: 10.1371/journal.pone.0146449 (PMC4704831; doi:10.1371/journal.pone.0146449)

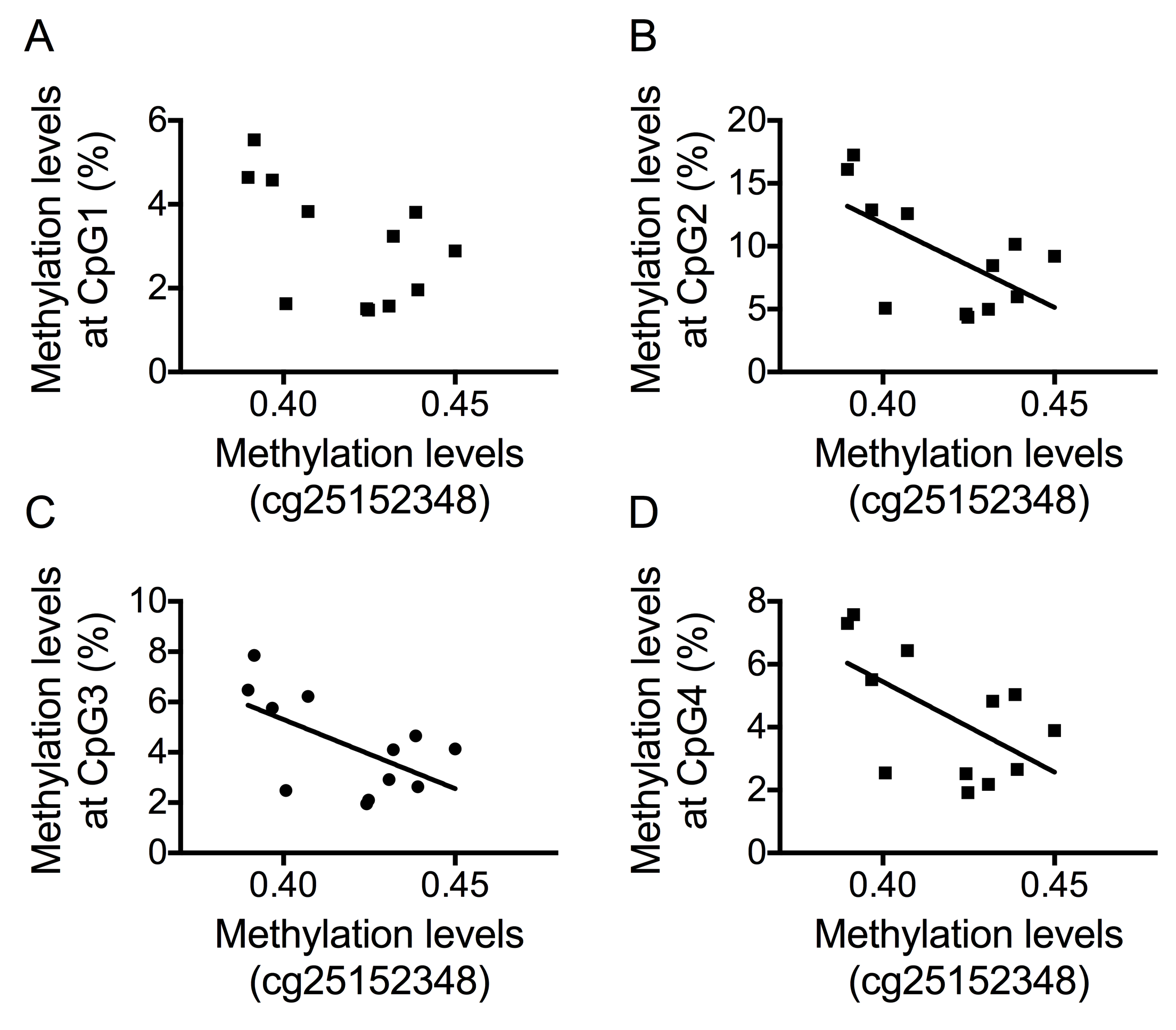

Supplement: S1 Fig — r = -0.54, P = 0.069 (A); r = -0.61, P = 0.037 * (B); r = -0.58, P = 0.046 * (C); r = -0.58, P = 0.047 * (D); * P < 0.05, Pearson's correlation coefficient. (TIFF) [file pone.0146449.s001.tiff]
