# Supplementary material for: Development of Biomarkers Based on DNA Methylation in the NCAPH2/LMF2 Promoter Region for Diagnosis of Alzheimer’s Disease and Amnesic Mild Cognitive Impairment
Source: PLoS One. 2016 Jan 7;11(1):e0146449. doi: 10.1371/journal.pone.0146449 (PMC4704831; doi:10.1371/journal.pone.0146449)
Supplement: S1 Table — Table indicates the target ID assigned by the Illumina Infinium HD Methylation Assay as well the gene name, accession number and gene group registered in UCSC. (DOCX) [file pone.0146449.s002.docx]

**S1 Table. 57 Candidate Loci Obtained by Illumina Infinium HD Methylation Assay.**

| **Target ID** | **UCSC REFGENE NAME** | **UCSC REFGENE ACCESSION** | **UCSC REFGENE GROUP** |
| --- | --- | --- | --- |
| cg00983705 | *ATXN1;ATXN1* | NM_001128164;NM_000332 | 5'UTR;5'UTR |
| cg01564925 | *BEST4* | NM_153274 | 1stExon |
| cg01582947 | *ZNF777* | NM_015694 | 5'UTR |
| cg01756799 | *COASY;COASY;COASY;COASY;COASY* | NM_025233;NM_001042529;NM_001042532;NM_001042530;NM_001042531 | 1stExon;Body;Body;Body;5'UTR |
| cg02174006 | *FAM190B* | NM_018999 | TSS200 |
| cg02944437 | *CDRT15* | NM_001007530 | TSS200 |
| cg03294491 | *SMAD2;SMAD2;SMAD2* | NM_005901;NM_001135937;NM_001003652 | TSS1500;TSS1500;TSS1500 |
| cg04174180 | *ZNF23* | NM_145911 | TSS1500 |
| cg04293460 | *GTF2A1;GTF2A1* | NM_015859;NM_201595 | TSS1500;TSS1500 |
| cg04552737 | *GLB1L;STK16;STK16* | NM_024506;NM_001008910;NR_026909 | 5'UTR;TSS1500;TSS1500 |
| cg04695796 | *CAMK1* | NM_003656 | 5'UTR |
| cg05304806 | *ZC3H14;ZC3H14;ZC3H14;ZC3H14;ZC3H14* | NM_207661;NM_001160104;NM_001160103;NM_207660;NM_024824 | 5'UTR;Body;Body;Body;Body |
| cg05411186 | *DHX30;DHX30;MIR1226* | NM_138615;NM_014966;NR_031595 | Body;Body;TSS200 |
| cg06638023 | *ZBTB45* | NM_032792 | 5'UTR |
| cg06671621 | *CLSTN3;RBP5* | NM_014718;NM_031491 | TSS1500;TSS200 |
| cg06695761 | *SGCE;PEG10;SGCE;PEG10;SGCE* | NM_001099401;NM_015068;NM_001099400;NM_001040152;NM_003919 | TSS1500;5'UTR;TSS1500;5'UTR;TSS1500 |
| cg07859880 | *ECH1* | NM_001398 | TSS1500 |
| cg08119452 | *OLFM4* | NM_006418 | TSS1500 |
| cg08727202 | *MPST;MPST;TST;MPST;MPST* | NM_001013436;NR_024038;NM_003312;NM_001130517;NM_021126 | TSS1500;TSS1500;Body;TSS1500;TSS1500 |
| cg09094393 | *FBXO30* | NM_032145 | TSS1500 |
| cg09564509 | *HDLBP;HDLBP* | NM_203346;NM_005336 | 5'UTR;5'UTR |
| cg09719956 | *SNORA1;SNORA8;SNORD6* | NR_003026;NR_002920;NR_003036 | TSS1500;TSS1500;TSS1500 |
| cg09898695 | *SPINT1;SPINT1;SPINT1* | NM_003710;NM_001032367;NM_181642 | TSS1500;TSS1500;TSS1500 |
| cg10523140 | *TBC1D8* | NM_001102426 | TSS1500 |
| cg10634115 | *UTS2R* | NM_018949 | TSS1500 |
| cg11641595 | *SERPINE2;SERPINE2;SERPINE2* | NM_006216;NM_001136530;NM_001136528 | 5'UTR;Body;5'UTR |
| cg12093180 | *ADNP;ADNP* | NM_015339;NM_181442 | TSS1500;TSS1500 |
| cg12556325 | *C7orf29;LRRC61;LRRC61* | NM_138434;NM_023942;NM_001142928 | TSS1500;5'UTR;5'UTR |
| cg13523072 | *COASY;COASY;COASY;COASY;COASY* | NM_025233;NM_001042529;NM_001042532;NM_001042530;NM_001042531 | 1stExon;Body;Body;Body;5'UTR |
| cg13645954 | *ASB10;ASB10;ASB10* | NM_001142459;NM_080871;NM_001142460 | TSS1500;TSS200;TSS1500 |
| cg13947830 | *MIB2;MIB2;MIB2;MIB2;MIB2;MIB2* | NM_001170687;NM_001170688;NM_080875;NM_001170689;NM_001170686;NR_033183 | Body;Body;Body;5'UTR;Body;Body |
| cg13976049 | *ZFP92* | NM_001136273 | TSS1500 |
| cg14559422 | *FAM54B;FAM54B;FAM54B;LOC646471;FAM54B* | NM_001099625;NM_001099627;NM_001099626;NR_024498;NM_019557 | 5'UTR;5'UTR;5'UTR;Body;5'UTR |
| cg14905466 | *SGK1;SGK1* | NM_001143677;NM_001143676 | TSS200;Body |
| cg16004008 | *NRGN;NRGN* | NM_006176;NM_001126181 | TSS1500;TSS1500 |
| cg16151082 | *PLEKHF2* | NM_024613 | TSS1500 |
| cg16618493 | *ZBTB7B* | NM_015872 | 5'UTR |
| cg17700453 | *XRRA1* | NM_182969 | 5'UTR |
| cg18865535 | *KIAA1026;KIAA1026;KIAA1026;KIAA1026;KIAA1026* | NM_201628;NM_001017999;NM_001018001;NM_015209;NM_001018000 | Body;5'UTR;5'UTR;Body;Body |
| cg18880660 | *F13A1* | NM_000129 | TSS1500 |
| cg19071452 | *NMI* | NM_004688 | TSS1500 |
| cg19205533 | *RERG* | NM_032918 | 5'UTR |
| cg19764096 | *C14orf184* | NM_001080113 | TSS200 |
| cg20246851 | *SEMA4B* | NM_020210 | 5'UTR |
| cg20315554 | *MSRB3;MSRB3* | NM_001031679;NM_198080 | 5'UTR;Body |
| cg21197871 | *C8orf38* | NM_152416 | TSS1500 |
| cg21556281 | *HOXA3;HOXA3;HOXA3* | NM_153631;NM_153632;NM_030661 | 5'UTR;TSS1500;5'UTR |
| cg22375763 | *LRG1* | NM_052972 | 1stExon |
| cg22531371 | *MUC1;MUC1;MUC1;MUC1;MUC1;MUC1;MUC1* | NM_001018016;NM_001044391;NM_001044393;NM_001044392;NM_001044390;NM_002456;NM_001018017 | TSS200;TSS200;TSS200;TSS200;TSS200;TSS200;TSS200 |
| cg22677858 | *IPO9* | NM_018085 | TSS1500 |
| cg23541619 | *SH3BP5* | NM_001018009 | TSS1500 |
| cg23779106 | *DUSP12* | NM_007240 | 1stExon |
| cg25152348 | *NCAPH2;LMF2;NCAPH2;NCAPH2;NCAPH2* | NM_152299;NM_033200;NM_152299;NM_014551;NM_014551 | 1stExon;TSS1500;5'UTR;5'UTR;1stExon |
| cg26812418 | *CPE* | NM_001873 | TSS200 |
| cg26963790 | *ZMYND11;ZMYND11;ZMYND11* | NM_212479;NM_001161482;NM_006624 | TSS1500;TSS1500;TSS1500 |
| cg26991433 | *RREB1;RREB1;RREB1;RREB1* | NM_001003700;NM_001003699;NM_001168344;NM_001003698 | 5'UTR;5'UTR;5'UTR;5'UTR |
| cg27173717 | *MFSD2A;MFSD2A* | NM_032793;NM_001136493 | TSS1500;TSS1500 |
