# Supplementary material for: Development of Biomarkers Based on DNA Methylation in the NCAPH2/LMF2 Promoter Region for Diagnosis of Alzheimer’s Disease and Amnesic Mild Cognitive Impairment
Source: PLoS One. 2016 Jan 7;11(1):e0146449. doi: 10.1371/journal.pone.0146449 (PMC4704831; doi:10.1371/journal.pone.0146449)
Supplement: S2 Table — * P < 0.05, Spearman's rank correlation coefficient. (DOCX) [file pone.0146449.s003.docx]

**S2 Table. Correlations between Methylation Level and Age, Duration of Disease or Age at Onset.**

| **CpG locus** | **Age** | | **Duration of disease** | | **Age at onset** | |
| --- | --- | --- | --- | --- | --- | --- |
|  | ***ρ*** | ***P*** | ***ρ*** | ***P*** | ***ρ*** | ***P*** |
| CpG 1 | -0.20 | 0.06 | 0.21 | 0.11 | -0.24 | 0.07 |
| CpG 2 | -0.20 | 0.07 | 0.19 | 0.16 | -0.20 | 0.14 |
| CpG 3 | -0.22 | 0.04 * | 0.03 | 0.83 | -0.19 | 0.16 |
| CpG 4 | -0.14 | 0.19 | 0.15 | 0.25 | -0.04 | 0.78 |
